# An Audit of the Prescribing and Monitoring of Antipsychotic Medication in an Older Adult Inpatient Psychiatric Ward Using NICE Guidance [CG178] Psychosis and Schizophrenia in Adults: Prevention and Management

**DOI:** 10.1192/bjo.2024.554

**Published:** 2024-08-01

**Authors:** Alaa Shabaka, Thomas Parry, Mary Ugah, Stephen De Souza

**Affiliations:** Somerset NHS Foundation Trust, Taunton, United Kingdom

## Abstract

**Aims:**

The National Institute for Health and Care Excellence (NICE) offers guidance for prescribing and monitoring of antipsychotic medications. In this audit we sought to investigate if our unit was compliant with this guidance.

**Methods:**

The audit was carried out on a 28 bedded older adult inpatient psychiatric unit. The notes of all patients admitted to this ward on 27/11/2023 were reviewed. Any patient on an antipsychotic was included in the audit. Four standards reflecting the prescribing and monitoring of antipsychotics were identified. These were:

1. 1.3.5.1 The choice of antipsychotic medication should be made by the service user and healthcare professional together, taking into account the views of the carer if the service user agrees.
2. 1.3.6.1 Before starting antipsychotic medication, undertake and record the baseline investigations.
3. 1.3.6.2 Before starting antipsychotic medication, offer the person with psychosis or schizophrenia an electrocardiogram (ECG).
4. 1.3.6.3 Treatment with antipsychotic medication should be considered an explicit individual therapeutic trial.
5. 1.3.6.4 Monitor and record the following (response to treatment – side effects – adherence – physical health) regularly and systematically throughout treatment.

These five areas of guidance were broken down into 22 domains which are outlined in results below.

**Results:**

Of 28 patients admitted to the ward, 22 were on antipsychotic medication.

1.3.5.1: Medication benefits were discussed and documented in 9/19 cases (47%), with 3 patients refusing to engage in this discussion. Side effects were discussed and documented in 5/21 cases (23%).

1.3.6.1: Patients underwent a range of investigations. In some cases, the patient hadn't been on the medication for long enough to require additional tests. Some patients were excluded as they refused testing. Glycosylated Haemoglobin (100%), Weight (100%), Pulse and Blood Pressure (100%), Blood Lipid Profile (86%), Prolactin Levels (77%), Assessment of nutritional status, diet (77%), baseline fasting blood glucose (38%), Level of Physical Activity (31%), Assessment of any movement disorder (22%), Waist Circumference (0%).

1.3.6.2: An ECG was offered in 94% of cases.

1.3.6.3: The rationale of continuing, changing or stopping the medication was recorded in 86% cases and no patients had antipsychotic doses above BNF maximum.

1.3.6.4: Overall physical health monitoring, weekly weights and, pulse and BP at 12 weeks (100%). Adherence and response to treatment were both 95%. Measurement of glycaemic control (57%), movement disorders (14%) and side effects (13%).

**Conclusion:**

While there are areas of good practice, there are a number of significant omissions. Remedies to these deficits will be proposed.

